# 超高效液相色谱-Orbitrap高分辨质谱用于减肥和壮阳类保健食品中32种非法添加药物的快速筛查和确证

**DOI:** 10.3724/SP.J.1123.2021.12009

**Published:** 2022-06-08

**Authors:** Hongbin XU, Shenping ZHANG, Ruyun DU, Jing ZHOU, Shiyu WENG

**Affiliations:** 上海市质量监督检验技术研究院, 国家市场监管重点实验室(乳及乳制品检测与监控技术), 上海 200233; Shanghai Institute of Quality Inspection and Technical Research, State Key Laboratory of Milk and Dairy Products Detection and Monitoring Technology for State Market Regulation, Shanghai 200233, China

**Keywords:** 超高效液相色谱, 高分辨质谱, 非法添加药物, 保健食品, 快速筛查, ultra-high performance liquid chromatography (UHPLC), high-resolution mass spectrometry (HRMS), illegally added drugs, health foods, rapid screening

## Abstract

建立了基于超高效液相色谱-Orbitrap高分辨质谱的快速筛查及确证减肥和壮阳类保健食品中32种非法添加药物的分析方法,并总结了数据库建立和应用的相关要点。研究对象聚焦于非法添加药物的衍生物,在对比正负离子模式下各化合物响应强度的基础上建立了高分辨质谱信息库,对提取溶剂、色谱柱温度等实验条件进行了详细探究,尽可能给出了较宽的标准曲线线性范围。使用Hypersil gold vanquish色谱柱(100 mm×2.1 mm, 1.9 μm),梯度洗脱,流量0.3 mL/min,正、负离子全扫描/数据依赖的二级扫描模式,在17 min内完成32种目标化合物的数据采集,通过TraceFinder软件进行快速定性筛查和定量。结果显示在17 min内32种化合物能得到较好分离;2种基质加标溶液中32种化合物的一级质谱离子精确质量数的实测值与理论值均在5×10^-6^误差之内,二级碎片离子质量数的实测值与理论值均在1×10^-5^误差之内;方法学验证结果表明,所有化合物均显示出优异的线性关系,相关系数(*r*^2^)均大于0.99;固体基质中除达泊西汀、羟基硫代豪莫西地那非、硫代豪莫西地那非、硫代西地那非、去甲基硫代西地那非的回收率较低外,其余27种化合物的回收率为50.5%~84.5%,相对标准偏差(RSD)为1.2%~13%,液体基质中32种化合物的回收率范围为60.4%~109.3%, RSD为0.77%~8.2%;在48份减肥及壮阳类保健食品中检出1份阳性样品,检出率2.08%。该方法操作简单,结果定性准确,可用于减肥及壮阳类保健食品中32种非法添加药物的快速筛查及确证。

## Abstract (en)

A novel method based on ultra-high performance liquid chromatography-orbitrap high-resolution mass spectrometry (UHPLC-Orbitrap HRMS) was developed for the rapid screening and confirmation of 32 illegally added drugs in slimming and anti-impotence health foods. In addition, the key points of the database establishment and application are summarized. This research focused on the derivatives of illegally added drugs. An HRMS database was established by comparing the response intensity of each compound in the positive and negative modes. The experimental conditions such as the type of extraction solvent and chromatographic column temperature were explored in detail. The analytes were separated on a Hypersil gold vanquish column (100 mm×2.1 mm, 1.9 μm) by gradient elution with acetonitrile/water (containing 0.1%(v/v) formic acid) as the mobile phase at a flow rate of 0.3 mL/min. Positive and negative ion full scanning/data-dependent secondary scanning mode was used to collect the 32 target compounds within 17 min, and TraceFinder software was used to screen the fragment ions. All the 32 compounds could be well separated within 17 min. The measured and theoretical values of the exact mass of the 32 compounds in the two matrix-spiked solutions were within an error of 5×10^-6^, and the MS^2^ fragment ions were within an error of 1×10^-5^. All the compounds showed an excellent linear relationship, with correlation coefficients (*r*^2^) above 0.99. Except dapoxetine, hydroxythiohomo sildenafil, thiohomo sildenafil, thiosildenafil, desmethyl thiosildenafil, the recoveries ranged from 50.5% to 84.5% in the solid matrix, with the relative standard deviations (RSDs) ranging from 1.2% to 13%. The recoveries were 60.4% to 109.3% in the liquid matrix, with the RSDs ranging from 0.77% to 8.2%. The matrix effect (ME) values of the 32 compounds ranged from 0.61 to 0.95 in the solid matrix and from 0.73 to 1.09 in the liquid matrix. Thiohomo sildenafil, desmethyl thiosildenafil, and chlorpretadalafil exhibited strong matrix inhibitory effects in the solid matrix. Therefore, solid and liquid negative matrix extracts were used to prepare a series of mixed standard solutions in order to reduce the ME values. The limits of detection (LODs) were 0.02 mg/kg for the 32 drugs in the liquid sample and 0.02 mg/kg for 29 compounds in the solid sample; the LODs for chlorothalidone, udenafil, and desmethyl thiosildenafil in the solid sample were 0.04 mg/kg. When the retention time in the self-built database matches the sample collection method, it should be used as one of the screening conditions. As for the selection of the matching mode, if the identify mode is selected, the retention time is a necessary condition for compound confirmation. When the retention time does not meet the requirements, subsequent screening of the fragment ions and isotope abundance ratios will not be performed. If the confirm mode is selected, the retention time is the optional condition for compound confirmation. When the retention time does not meet these requirements, subsequent matching of other conditions such as fragment ions and isotope information is required. Isotope information is very important in HRMS and is an effective supplement to the first-order extracted mass. Therefore, its use is recommended, but the isotope abundance ratio will be even lower when the target content is very low in the complex matrix, which may affect isotope matching. In addition, if the fragment ions are not detected in the screening results of the TraceFinder software but can be extracted in the data browser, their intensity threshold in the screening conditions can be further reduced to find the corresponding fragment ions. One positive sample was detected among 48 healthy food samples, with a detection rate of 2.08%. This method has the advantages of simple operation and high accuracy. It can be used for the rapid screening and confirmation of 32 illegally added drugs in slimming and anti-impotence health foods.

近年来,人们对保健食品的需求与日俱增,尤其是减肥和壮阳类产品格外受到人们的青睐,然而该类产品检出非法添加药物的情况屡见不鲜。不法商家为了达到产品宣称的保健功效,在产品中超量、超范围添加处方药及其类似物或者衍生物,给消费者的身体健康造成了隐患。为严厉打击非法添加行为、保护消费者身体健康,需要开发快速准确的检测方法用于减肥及壮阳类非法添加药物的检测。

目前常用的非法添加检验方法主要有薄层色谱法^[1⇓-3]^、液相色谱法^[4⇓-6]^、常规液相色谱-质谱联用法^[7⇓-9]^,这些方法大都存在选择性差、干扰多、定性不准确等缺点。作为质谱分析器的突破性技术,Orbitrap通过静电场轨道阱的方式提供高分辨率及精确离子质量数,最高分辨率可达240000 FWHM (full width at half maxima)以上。目前已有报道使用超高效液相色谱-Orbitrap高分辨质谱对保健食品中非法添加药物进行检测。谭会洁等^[10]^报道了保健食品中15种减肥类化合物的超高效液相色谱-高分辨质谱筛查方法,建立了筛查数据库,采用Hypersil gold C18色谱柱分离,正负离子模式同时扫描,回收率达到79.7%~95.4%,但未就数据库应用时各项参数的意义进行说明。黄泽玮等^[11]^建立了基于超高效液相色谱-Orbitrap-Elite高分辨质谱的快速筛查及确证减肥及壮阳类保健食品和中成药中54种非法添加物的方法,采用Hypersil gold C18色谱柱在28 min内完成54种目标化合物的分离,全扫描/数据依赖的二级扫描(Full MS/dd-MS^2^)模式完成高精度一级、二级质谱信息采集,但在建立数据库时仅涉及正离子模式。洪灯等^[12]^针对不法商家用非法添加药物的类似物来规避法定方法检测的现象,开展了超高效液相色谱-Orbitrap高分辨质谱法筛查保健食品中西布曲明及其衍生物的检测方法,定量限为25 μg/kg,加标回收率为93.5%~103.5%,但标准曲线线性范围仅为0.5~20 μg/L,对于高浓度的检出值需要多次稀释至标准曲线浓度中值附近进行测定,容易引入不确定度因子。我国现行的保健食品中非法添加药物的检测方法有BJS 201710《保健食品中75种非法添加化学药物的检测》和BJS 201805《食品中那非类物质的测定》,覆盖了绝大部分可能的非法添加药物,但随着药物研发的推进,非法添加药物的衍生物及类似物日益增多,也应将其纳入检测范围。

本文将Orbitrap高分辨质谱用于减肥及壮阳类保健食品中32种非法添加药物的快速筛查及确证,在对比正负离子模式下各化合物响应强度的基础上建立了高分辨质谱信息库,总结了数据库建立及筛查数据分析的要点,对提取溶剂、色谱柱温度等实验条件进行了详细探究,尽可能给出了较宽的标准曲线线性范围。通过Full MS/dd-MS^2^模式,在17 min内完成了目标物的分离和碎片离子质量数的采集,并通过TraceFinder软件完成数据分析。本方法具有快速、准确、灵敏度高的特点,有助于打击保健食品的非法添加行为。

## 1 实验部分

### 1.1 仪器、试剂与材料

Thermo Q Exactive PLUS超高效液相色谱-质谱联用系统包括vanquish VF-P10-A液相色谱系统及Orbitrap高分辨质谱;Xcalibur 4.3软件用于质谱仪器控制及数据采集;TraceFinder 5.0软件用于数据分析;电子天平(感量0.1 mg, MS204S,德国Mettler Toledo公司);离心机(Centrifuge 5804);涡旋振荡器(Reax Control,德国Heidolph);超纯水器(美国Millipore)。

大黄素(emodin,纯度>98.0%)、他达拉非甲基氯化物(chlorpretadalafil,纯度>98.0%)以及其余30种非法添加药物对照品溶液(100 mg/L,甲醇)购自上海普誉科贸有限公司。HPLC级甲醇(纯度>99.9%)购自赛默飞世尔科技(中国)有限公司,HPLC级乙醇(纯度>99.9%)购自默克股份两合公司,甲酸(分析纯)购自上海阿拉丁生化科技股份有限公司。

48份保健食品(片剂、硬胶囊、粉剂、口服液、酒类)来自上海市质量监督检验技术研究院监督抽样。样品种类包括灵芝孢子粉、氨基酸口服液、保健酒类、西洋参含片及胶囊,基质成分较为复杂,主要成分为天然成分、羧甲基纤维素钠、淀粉、蔗糖、葡萄糖。阴性样品均属于监督抽检项目中按照国家标准方法未检出的样品,随机挑选未检出的片剂、粉剂、硬胶囊(内容物)样品混合均匀,作为阴性固体样品基质;挑选未检出的口服液、酒类样品混合均匀,作为阴性液体样品基质。

### 1.2 对照品溶液的配制

称取适量大黄素和他达拉非甲基氯化物对照品,分别用乙醇和50%(v/v,下同)甲醇水溶液配制成1000 mg/L的储备液,再分别用甲醇稀释至100 mg/L;分别取32种对照品溶液,以甲醇稀释为5 mg/L的混合对照品溶液Ⅰ,再以甲醇稀释为500 μg/L的混合对照溶液Ⅱ。上述溶液均保存在-20 ℃冰箱,临用前取出恢复至室温。

取混合对照品溶液Ⅱ,以阴性基质提取液逐级稀释为1.0、2.0、5.0、10.0、50.0、100.0、200.0 μg/L的系列对照品标准溶液。

### 1.3 供试品溶液的制备

片剂研细后备用,硬胶囊取内容物粉末备用,粉剂、口服液、酒类可直接称取。精密称定0.5 g样品置于15 mL具塞离心管中,加入10 mL 50%甲醇水溶液,振荡混合均匀,25 ℃下恒温超声提取15 min,超声功率53 kHz,于5000 r/min离心2 min,上清液过0.22 μm微孔滤膜,滤液即为供试品溶液。对于浓度超过标准曲线上限的待测液,需用50%甲醇水溶液适当稀释后再次测定。

### 1.4 仪器条件

#### 1.4.1 色谱

色谱柱为Thermo Hypersil gold vanquish (100 mm×2.1 mm, 1.9 μm),柱温:25 ℃,流速:0.3 mL/min,流动相A: 0.1%甲酸水溶液,流动相B: 0.1%甲酸乙腈溶液,梯度洗脱程序:0~2 min, 10%B; 2~8 min, 10%B~90%B; 8~10 min, 90%B; 10~11 min, 90%B~10%B; 11~14 min, 10%B。进样量:5 μL。

#### 1.4.2 质谱

离子源:可加热电喷雾离子源(HESI);离子源温度:325 ℃;喷雾电压:3.50 kV(正离子模式), 2.8 kV(负离子模式);透镜电压:50.0 V;鞘气流速:40 arb;辅助气流速:10 arb;辅助气温度:350 ℃;扫描模式:Full MS/dd-MS^2^;采集范围:*m/z* 100.0~1000.0;一级质谱分辨率为70000 FWHM; 自动增益控制目标离子数(AGC target): 1×10^6^; C-trap最大注入时间:200 ms;二级质谱分辨率17500 FWHM; AGC target: 2×10^5^; C-trap最大注入时间:60 ms;归一化碰撞能(NCE): 20%/40%/60%;动态排除:8.0 s。各个化合物的质谱分析参数见表1。

**表1:** 32种化合物的UHPLC-HRMS分析参数

No.	Compound			Formula		Measured mass (*m/z*)		Fragments (*m/z*)		*t*_R_/min	Adduct
1	avanafil (阿伐那非)			C_23_H_26_ClN_7_O_3_		484.18585		375.12159, 233.10332		6.00	[M+H]^+^
2	acetilacid (那非乙酰酸)			C_18_H_20_N_4_O_4_		357.15515		329.12390, 300.08511		6.85	[M+H]^+^
3	dapoxetine (达泊西汀)			C_21_H_23_NO		306.18439		261.12695, 183.08032		6.67	[M+H]^+^
4	xanthoanthrafil (苯酰胺那非)			C_19_H_23_N_3_O_6_		390.16553		151.07516, 135.04373		6.95	[M+H]^+^
5	piperiacetidenafil (苯噻啶红地那非)			C_24_H_31_N_5_O_3_		438.24939		297.12422, 341.16089		5.93	[M+H]^+^
6	benzocaine (苯佐卡因)			C_9_H_11_NO_2_		166.08601		138.05492, 120.04429		6.31	[M+H]^+^
7	bendroflumethiazide (苄氟噻嗪)			C_15_H_14_F_3_N_3_O_4_S_2_		420.03149		289.04599, 327.96851		7.13	[M-H]^-^
8	yohimbine (育亨宾)			C_21_H_26_N_2_O_3_		355.20090		212.12802, 144.08075		5.43	[M+H]^+^
9	hydroxyacetildenafil (羟基红地那非)			C_25_H_34_N_6_O_4_		483.27103		297.13419, 127.08654		5.73	[M+H]^+^
10	hydroxythiohomo sildenafil (羟基硫代豪莫西地那非)			C_23_H_32_N_6_O_4_S_2_		521.19958		299.09589, 327.12759		6.74	[M+H]^+^
11	hydroxyvardenafil (羟基伐地那非)			C_23_H_32_N_6_O_5_S		505.22235		344.14746, 169.09691		5.73	[M+H]^+^
12	thiohomo sildenafil (硫代豪莫西地那非)			C_23_H_32_N_6_O_3_S_2_		505.20401		299.09607, 113.10741		6.92	[M+H]^+^
13	thiosildenafil (硫代西地那非)			C_22_H_30_N_6_O_3_S_2_		491.18918		341.14389, 407.12027		6.82	[M+H]^+^
14	*N*-desmethylsibutramine hydrochloride			C_16_H_24_ClN·HCl		266.16666		125.01524, 139.03091		6.72	[M+H]^+^
	(盐酸*N*-单去甲基西布曲明)										
15	*N*,*N*-didesmethylsibutramine hydrochloride			C_15_H_22_ClN·HCl		252.15092		125.01521, 139.03085		6.69	[M+H]^+^
	(盐酸*N*,*N*-双去甲基西布曲明)										
16	chlorthalidone (氯噻酮)			C_14_H_11_CLN_2_O_4_S		337.00583		146.02345, 189.97272		5.70	[M-H]^-^
17	lorcaserin (氯卡色林)			C_11_H_14_ClN		196.08885		179.06218, 167.06212		5.52	[M+H]^+^
18	hydrochlorothiazide (氢氯噻嗪)			C_7_H_8_ClN_3_O_4_S_2_		295.95804		268.94708, 204.98419		3.06	[M-H]^-^
19	torasemide (托拉塞米)			C_16_H_20_N_4_O_3_S		347.11835		262.06564, 195.07954		6.04	[M-H]^-^
20	gendenafil (庆地那非)			C_19_H_22_N_4_O_3_		355.17612		327.14514, 285.13467		7.44	[M+H]^+^
21	emodin (大黄素)			C_15_H_10_O_5_		269.04623		225.05583, 241.05090		8.11	[M-H]^-^
22	indapamide (吲达帕胺)			C_16_H_16_O_3_N_3_ClS		366.06686		132.08073, 117.05728		6.75	[M+H]^+^
23	desmethyl sildenafil (去甲基西地那非)			C_21_H_28_N_6_O_4_S		461.19626		283.11868, 255.12376		5.97	[M+H]^+^
24	desmethyl thiosildenafil (去甲基硫代西地那非)			C_21_H_28_N_6_O_3_S_2_		477.17288		299.09586, 315.09033		6.79	[M+H]^+^
25	nortadalafil (去甲他达拉非)			C_21_H_17_N_3_O_4_		376.12857		262.08588, 254.09195		6.69	[M+H]^+^
No.	Compound			Formula		Measured mass (*m/z*)		Fragments (*m/z*)		*t*_R_/min	Adduct
26	*N*-desethyl acetildenafil (去乙基红地那非)			C_23_H_30_N_6_O_3_		439.24466		297.13419, 313.12927		5.78	[M+H]^+^
27	*N*-desethyl vardenafil (去乙基伐地那非)			C_21_H_28_N_6_O_4_S		461.19577		344.14749, 299.11346		5.97	[M+H]^+^
28	rimonabant (利莫那班)			C_22_H_21_Cl_3_N_4_O		463.08517		380.99548, 299.01343		9.08	[M+H]^+^
29	lidocaine (利多卡因)			C_14_H_22_N_2_O		235.18016		86.09662, 58.06575		4.33	[M+H]^+^
30	chlorpretadalafil (他达拉非甲基氯化物)			C_22_H_19_ClN_2_O_5_		337.00583		146.02345, 189.97272		7.91	[M+H]^+^
31	dimethylsildenafil (二甲基西地那非)			C_23_H_32_N_6_O_4_S		489.22803		283.11920, 344.14856		6.14	[M+H]^+^
32	udenafil (乌地那非)			C_25_H_36_N_6_O_4_S		517.25854		283.11874, 299.11380		6.22	[M+H]^+^

#### 1.4.3 定性筛查条件

目标峰面积阈值设置为50000,以排除部分杂峰的影响,减少筛查时间,信噪比阈值设置为10,精确质量数偏差设置为5×10^-6^;保留时间的判定模式设置为confirm,保留时间窗口宽度为30 s;碎片离子判定模式设置为confirm,最小匹配度为1个碎片离子,碎片离子强度阈值为5000,碎片离子质量数偏差为1×10^-5^;同位素筛查可作为目标峰的补充筛查信息,启用时判定模式为confirm,匹配度阈值为90%,质量数偏差为1×10^-5^,强度偏差为20%。

## 2 结果与讨论

### 2.1 实验条件考察

参考相关文献^[10,12]^,分别考察了甲醇、乙腈、50%甲醇水溶液、50%乙腈水溶液对基质加标样品中32种化合物的超声提取效果。结果表明,以50%甲醇水溶液进行提取,32种化合物在固体基质和液体基质中的回收率均高于其他3种提取溶剂,其中那非乙酰酸在3个水平(0.2、0.6、1.0 mg/kg)下的加标回收率平均值分别由42.8%、52.1%、66.7%提高到70.2%、88.3%、92.4%,苯佐卡因的加标回收率平均值由34.1%、39.4%、48.4%提高到78.6%、91.2%、109.3%,氯卡色林的加标回收率平均值由36.3%、49.5%、64.6%提高到76.1%、88.5%、94.4%。将超声处理时间由15 min延长至20 min,提取效果没有明显增加。考虑到节省样品前处理时间,本实验选择50%甲醇水溶液作为提取溶剂,超声提取15 min。

为选择合适的流动相,本实验对比了0.1%甲酸甲醇-0.1%甲酸水、10 mmol/L乙酸铵乙腈-0.1%甲酸水和0.1%甲酸乙腈-0.1%甲酸水作为流动相时的分离效果。结果表明,0.1%甲酸甲醇-0.1%甲酸水流动相体系中,去乙基伐地那非、去甲基西地那非、羟基伐地那非均在7.27 min出峰,未能完全分开;10 mmol/L乙酸铵乙腈-0.1%甲酸水体系中,去甲他达拉非、羟基硫代豪莫西地那非的响应值降低1个数量级,苯佐卡因的响应值降低2个数量级。相比而言,0.1%甲酸乙腈-0.1%甲酸水体系可获得最佳的色谱峰形和分离效果,不同化合物在合适的正负离子模式下均能获得较高的响应值。

柱温对分离效果也有一定的影响,分别考察了25、30、35 ℃下的分离情况,发现提高柱温至35 ℃时,去甲基硫代西地那非在其保留时间附近存在干扰峰,影响峰积分,同时各化合物保留时间减小,如达泊西汀的保留时间由6.67 min前移至6.61 min,保留时间整体偏移较小,在30 s的保留时间判定窗口中仍可实现目标峰的判定。另外部分化合物出现了多次保留的情况,如盐酸*N*,*N*-双去甲基西布曲明在6.30 min和6.59 min出现了两次保留,乌地那非在5.80 min和6.16 min出现了两次保留。相比而言,25 ℃柱温下,各化合物峰形对称,去甲基硫代西地那非与基质中的干扰物质可完全分离,有利于峰积分。因此确定最佳柱温为25 ℃。

综上,最终确定了1.4节的色谱条件,加标量为1.0 mg/kg的阴性样品提取液(供试品溶液质量浓度50 μg/L)中各目标化合物的提取离子色谱图见图1。在现有色谱条件下,氢氯噻嗪、羟基伐地那非、去乙基红地那非保留时间在3~6 min内的峰形并不是非常理想,这是因为供试品溶液的溶剂(50%甲醇水)与流动相(0.1%甲酸乙腈-0.1%甲酸水)不一致而产生了轻微的溶剂效应,但其余29种化合物峰形良好、基线干净。为了探究该现象是否会影响低浓度下这3种化合物的定性定量检测,调整供试品溶液的浓度为10 μg/L(基质加标水平0.2 mg/kg),测试结果表明这3种化合物的响应很强,峰面积在3×10^6^~6×10^6^之间,且在保留时间附近的基线非常干净,说明轻微的溶剂效应并不影响低浓度下这3种化合物定性和定量检测的准确性。

**图1:**
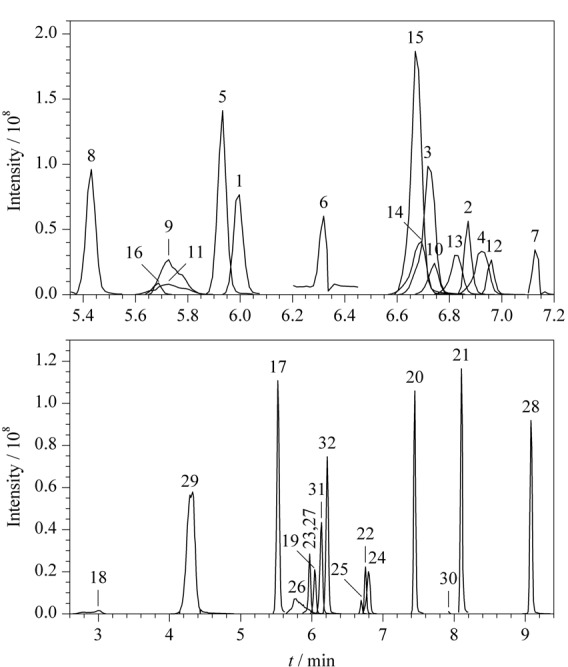
32种化合物的提取离子色谱图(加标量1.0 mg/kg)

### 2.2 基质效应

固体基质包括灵芝孢子粉、西洋参含片及硬胶囊,主要成分是天然成分、羧甲基纤维素钠、淀粉、蔗糖,液体基质包括氨基酸口服液及酒类,主要成分为糖类、氨基酸,这些成分会干扰目标化合物的分离和检测^[13⇓-15]^。以基质匹配标准工作曲线的斜率与纯溶剂标准工作曲线的斜率的比值考察基质效应(matrix effect, ME), ME值小于0.80或者大于1.10,存在强基质效应,反之基质效应较弱。结果表明32种化合物在固体基质中的ME值为0.61~0.95,其中硫代豪莫西地那非、去甲基硫代西地那非、他达拉非甲基氯化物的ME值较低,分别为0.61、0.70、0.63;在液体基质中的ME值为0.73~1.09,其中羟基红地那非、硫代豪莫西地那非的ME值为0.73、0.75;可见部分化合物在两种基质中存在不同程度的基质效应,因此分别使用固体阴性基质和液体阴性基质提取液配制系列混合标准溶液,降低基质效应。

### 2.3 方法学考察

#### 2.3.1 线性范围和检出限

分别将两种基质提取液配制的系列混合标准溶液在最优条件下进样,每个浓度平行测定3次,以平均峰面积(*y*)对其质量浓度(*x*, μg/L)绘制标准曲线,得到相关系数(*r*^2^)。所有的化合物在合适的浓度范围内线性关系良好,*r*^2^均大于0.99。在空白基质样品中进行低浓度加标试验,计算信噪比,当供试品溶液的质量浓度为1 μg/L时(氯噻酮、去甲基硫代西地那非供试品溶液质量浓度为2 μg/L),有23种化合物的信噪比显示为“INF”,其余9种化合物的信噪比在50~240之间。若以3倍信噪比(*S/N*=3)对应的浓度计算检出限,则该值远低于现有值,不具备实际意义,因此本研究以1 μg/L计算检出限,结果见表2。标准曲线线性范围最低点为5 μg/L,最高浓度点根据化合物不同而分别为200、500 μg/L;液体样品中32种化合物的检出限为0.02 mg/kg,固体样品中29种化合物的检出限为0.02 mg/kg,氯噻酮、去甲基硫代西地那非和乌地那非的检出限为0.04 mg/kg。以标准曲线最低浓度点计算定量限为0.1 mg/kg。

**表2:** 32种化合物的线性方程、线性范围、相关系数和检出限

No.	Compound	Solid sample					Liquid sample				
		Linear equation	Linear range^*^	*r*^2^	LOD/ (mg/kg)		Linear equation		Linear range^*^	*r*^2^	LOD/ (mg/kg)
1	avanafil	*y*=2.41×10^6^*x*+4.55×10^7^	5-500	0.9924	0.02		*y*=2.13×10^6^*x*+3.66×10^7^		5-500	0.9941	0.02
2	acetilacid	*y*=2.20×10^6^*x*+4.16×10^6^	5-200	0.9985	0.02		*y*=2.03×10^6^*x*+2.14×10^7^		5-500	0.9970	0.02
3	dapoxetine	*y*=5.47×10^6^*x*+4.90×10^7^	5-500	0.9972	0.02		*y*=5.00×10^6^*x*+7.34×10^7^		5-500	0.9960	0.02
4	xanthoanthrafil	*y*=5.76×10^5^*x*+7.49×10^6^	5-500	0.9917	0.02		*y*=5.22×10^5^*x*+4.97×10^6^		5-500	0.9977	0.02
5	piperiacetidenafil	*y*=3.00×10^6^*x*+8.73×10^6^	5-200	0.9988	0.02		*y*=2.81×10^6^*x*+6.18×10^7^		5-500	0.9922	0.02
6	benzocaine	*y*=4.12×10^6^*x*-9.63×10^6^	5-200	0.9914	0.02		*y*=3.74×10^6^*x*+3.60×10^7^		5-500	0.9970	0.02
7	bendroflumethiazide	*y*=1.15×10^6^*x*+1.90×10^6^	5-500	0.9943	0.02		*y*=1.01×10^6^*x*+1.52×10^7^		5-500	0.9951	0.02
8	yohimbine	*y*=2.81×10^6^*x*+1.01×10^6^	5-200	0.9952	0.02		*y*=6.53×10^6^*x*+3.99×10^6^		5-200	0.9982	0.02
9	hydroxyacetildenafil	*y*=1.29×10^6^*x*+1.55×10^5^	5-200	0.9983	0.02		*y*=9.64×10^5^*x*+7.38×10^5^		5-200	0.9952	0.02
10	hydroxythiohomo sildenafil	*y*=1.38×10^6^*x*+2.81×10^6^	5-200	0.9962	0.02		*y*=7.16×10^5^*x*+5.72×10^6^		5-500	0.9978	0.02
11	hydroxyvardenafil	*y*=3.93×10^5^*x*+4.73×10^5^	5-200	0.9976	0.02		*y*=9.49×10^5^*x*+3.17×10^5^		5-200	0.9932	0.02
12	thiohomo sildenafil	*y*=2.27×10^6^*x*+4.87×10^6^	5-200	0.9938	0.02		*y*=1.35×10^6^*x*+1.45×10^7^		5-500	0.9942	0.02
13	thiosildenafil	*y*=2.25×10^6^*x*-3.53×10^6^	5-200	0.9995	0.02		*y*=1.03×10^6^*x*+1.31×10^7^		5-500	0.9943	0.02
14	*N*-desmethylsibutramine	*y*=2.82×10^6^*x*+1.10×10^6^	5-200	0.9975	0.02		*y*=3.38×10^6^*x*+1.48×10^7^		5-200	0.9945	0.02
	hydrochloride										
15	*N*,*N*-didesmethylsibutramine	*y*=1.27×10^6^*x*+1.05×10^6^	5-200	0.9982	0.02		*y*=1.43×10^6^*x*+5.65×10^6^		5-200	0.9904	0.02
	hydrochloride										
16	chlorthalidone	*y*=2.24×10^5^*x*+2.05×10^5^	5-200	0.9966	0.04		*y*=3.20×10^5^*x*+2.39×10^6^		5-500	0.9983	0.02
17	lorcaserin	*y*=2.98×10^6^*x*+3.97×10^6^	5-200	0.9945	0.02		*y*=3.03×10^6^*x*+1.11×10^7^		5-200	0.9946	0.02
18	hydrochlorothiazide	*y*=3.00×10^5^*x*-1.73×10^5^	5-200	0.9997	0.02		*y*=2.98×10^5^*x*+1.04×10^6^		5-200	0.9943	0.02
19	torasemide	*y*=9.78×10^5^*x*+2.87×10^6^	5-500	0.9983	0.02		*y*=8.06×10^5^*x*+1.05×10^6^		5-500	0.9964	0.02
20	gendenafil	*y*=3.63×10^6^*x*+2.27×10^7^	5-500	0.9985	0.02		*y*=3.29×10^6^*x*+4.60×10^7^		5-500	0.9949	0.02
21	emodin	*y*=2.60×10^6^*x*+2.51×10^6^	5-200	0.9916	0.02		*y*=2.96×10^6^*x*+1.20×10^6^		5-200	0.9926	0.02
22	indapamide	*y*=5.13×10^5^*x*+6.57×10^6^	5-500	0.9921	0.02		*y*=4.69×10^5^*x*+3.35×10^6^		5-500	0.9986	0.02
23	desmethyl sildenafil	*y*=1.12×10^6^*x*+1.76×10^6^	5-200	0.9940	0.02		*y*=8.79×10^5^*x*+5.67×10^6^		5-200	0.9904	0.02
24	desmethyl thiosildenafil	*y*=1.02×10^6^*x*+2.03×10^6^	5-500	0.9985	0.04		*y*=7.90×10^5^*x*+7.91×10^6^		5-500	0.9979	0.02
25	nortadalafil	*y*=4.84×10^6^*x*-1.01×10^6^	5-500	1	0.02		*y*=4.35×10^5^*x*+3.97×10^6^		5-500	0.9974	0.02
26	*N*-desethyl acetildenafil	*y*=7.81×10^5^*x*-4.59×10^5^	5-200	0.9983	0.02		*y*=7.06×10^5^*x*+7.71×10^5^		5-200	0.9962	0.02
27	*N*-desethyl vardenafil	*y*=6.65×10^6^*x*+1.03×10^6^	5-200	0.9967	0.02		*y*=8.87×10^5^*x*+5.66×10^6^		5-200	0.9904	0.02
28	rimonabant	*y*=2.46×10^6^*x*+2.68×10^6^	5-200	0.9953	0.02		*y*=2.74×10^6^*x*+2.04×10^6^		5-200	0.9981	0.02
29	lidocaine	*y*=5.97×10^6^*x*+1.78×10^7^	5-200	0.9954	0.02		*y*=5.42×10^6^*x*+2.67×10^7^		5-200	0.9929	0.02
30	chlorpretadalafil	*y*=1.81×10^5^*x*+5.23×10^4^	5-500	0.9998	0.02		*y*=1.67×10^5^*x*+8.56×10^5^		5-500	0.9990	0.02
31	dimethylsildenafil	*y*=1.84×10^6^*x*+8.15×10^6^	5-200	0.9946	0.02		*y*=1.16×10^6^*x*+2.08×10^7^		5-500	0.9917	0.02
32	udenafil	*y*=4.67×10^6^*x*+1.09×10^6^	5-200	0.9982	0.04		*y*=1.51×10^6^*x*+4.89×10^7^		5-200	0.9940	0.02

*y*: peak area; *x*: mass concentration, μg/L; * μg/L.

#### 2.3.2 重复性

各取6份固体基质和液体基质进行加标试验,加标水平为0.2 mg/kg,每份样品平行测定3次,取结果平均值计算相对标准偏差(RSD),考察重复性。结果表明32种化合物峰面积的RSD为0.35%~3.1%,均小于5%,说明该测定方法重复性良好。

#### 2.3.3 精密度

取一份加标样品溶液在一日内的不同时间点(共6个时间点,间隔4 h)测定,计算RSD值,记为日内精密度;在不同日期(连续5 d内的同一时间)测定,计算RSD值,记为日间精密度。32种化合物的日内精密度均小于6.5%,日间精密度均小于10%,说明测试方法精密度良好。

#### 2.3.4 稳定性

按照1.2节和1.3节分别制备对照品溶液和样品溶液,同一份对照品溶液和阳性样品溶液在0、2、4、6、8 h进样,计算RSD值,考察稳定性。标准溶液中32种化合物的峰面积RSD为0.42%~2.7%,均小于3%,表明32种化合物在测定时间8 h内稳定性良好。大黄素阳性样品溶液在8 h内进样5次,大黄素峰面积的RSD为1.4%,说明样品提取液在测定时间内非常稳定。

#### 2.3.5 回收率

精密称取两种阴性基质,分别加入5 mg/L的混合标准储备液20、60、100 μL,按照1.3节的方法进行样品处理,对应的加标水平为0.2、0.6、1.0 mg/kg。平行测定6次,计算回收率平均值和RSD,结果见表3。结果表明,固体基质中除达泊西汀、羟基硫代豪莫西地那非、硫代豪莫西地那非、硫代西地那非、去甲基硫代西地那非的回收率较低外,其余27种化合物的回收率为50.5%~84.5%,RSD为1.2%~13%,液体基质中32种化合物的回收率范围为60.4%~109.3%, RSD为0.77%~8.2%。

**表3:** 不同阴性基质中3个添加水平下的32种化合物的回收率及相对标准偏差(*n*=6)

No.	Compound	Recoveries (RSDs) in solid sample/%				Recoveries (RSDs) in liquid sample/%			
		0.2 mg/kg	0.6 mg/kg	1.0 mg/kg		0.2 mg/kg		0.6 mg/kg	1.0 mg/kg
1	avanafil	74.2(2.2)	53.9(5.2)	74.7(10)		72.5(2.5)		85.7(3.4)	86.5(5.2)
2	acetilacid	78.6(1.8)	82.7(2.8)	78.3(5.1)		70.2(3.8)		88.3(2.0)	92.4(2.1)
3	dapoxetine	50.5(1.5)	36.9(3.1)	49.9(4.1)		71.8(2.1)		96.1(2.0)	96.9(3.1)
4	xanthoanthrafil	69.9(4.8)	54.5(3.7)	75.3(4.2)		77.6(3.4)		98.7(2.0)	94.4(1.2)
5	piperiacetidenafil	72.7(2.0)	76.9(2.6)	74.6(7.8)		88.2(4.3)		92.1(5.0)	94.9(6.0)
6	benzocaine	84.3(12)	73.4(5.1)	70.0(3.0)		78.5(6.4)		91.2(8.2)	109.3(5.2)
7	bendroflumethiazide	77.5(4.0)	81.2(3.2)	73.1(3.7)		75.0(5.3)		84.3(2.5)	86.3(3.6)
8	yohimbine	74.3(2.4)	77.0(3.8)	78.7(6.0)		66.6(2.5)		86.7(2.7)	94.1(2.1)
9	hydroxyacetildenafil	71.6(2.9)	76.4(3.4)	70.4(7.4)		62.1(3.4)		77.1(1.5)	83.4(2.8)
10	hydroxythiohomo sildenafil	69.7(3.2)	41.0(3.7)	59.2(9.3)		77.2(4.3)		91.3(2.1)	85.9(1.6)
11	hydroxyvardenafil	67.6(3.4)	70.9(3.4)	67.8(8.5)		71.2(2.9)		82.2(1.5)	87.7(2.9)
12	thiohomo sildenafil	49.8(3.2)	23.4(3.1)	36.7(9.2)		64.9(5.1)		86.7(2.6)	88.5(2.2)
13	thiosildenafil	64.3(5.0)	36.5(2.6)	53.2(10)		74.3(4.4)		93.4(1.6)	94.0(1.7)
14	*N*-desmethylsibutramine hydrochloride	69.6(2.2)	74.2(2.7)	70.7(3.0)		67.7(2.8)		88.1(2.4)	98.1(1.2)
15	*N*,*N*-didesmethylsibutramine hydrochloride	66.2(3.3)	81.2(2.8)	70.1(5.0)		60.4(1.1)		83.1(2.5)	90.5(1.0)
16	chlorthalidone	84.1(5.8)	59.6(5.9)	65.7(5.1)		80.5(3.2)		102.0(3.0)	99.3(2.5)
17	lorcaserin	72.6(2.1)	75.8(3.6)	74.5(5.4)		76.0(1.6)		88.5(1.6)	94.4(2.1)
18	hydrochlorothiazide	74.1(7.4)	83.7(2.0)	73.3(4.3)		78.6(2.0)		88.2(1.3)	89.6(0.99)
19	torasemide	78.6(5.5)	84.5(3.8)	77.1(5.0)		73.3(4.5)		93.3(3.8)	92.1(2.3)
20	gendenafil	80.3(1.2)	54.3(3.9)	73.5(5.5)		85.3(3.7)		107.3(1.2)	100.0(2.3)
21	emodin	64.5(2.4)	73.0(3.1)	66.7(2.4)		81.6(4.5)		86.8(3.0)	90.2(2.5)
22	indapamide	78.7(5.1)	80.4(1.4)	77.5(8.1)		78.8(3.0)		91.3(1.9)	91.9(2.1)
23	desmethyl sildenafil	68.5(4.5)	74.6(3.4)	70.5(8.4)		74.6(2.5)		87.2(0.77)	90.3(2.8)
24	desmethyl thiosildenafil	45.8(6.2)	24.9(3.2)	38.3(11)		71.4(3.2)		95.9(2.9)	93.2(2.3)
25	nortadalafil	69.2(13)	71.9(5.5)	71.9(8.6)		68.4(7.9)		95.0(4.7)	91.4(3.0)
26	*N*-desethyl acetildenafil	76.4(2.6)	78.8(1.7)	74.2(8.2)		76.3(3.1)		88.0(1.2)	91.2(1.4)
27	*N*-desethyl vardenafil	68.5(4.5)	74.6(3.4)	70.5(10)		74.6(2.5)		87.2(0.78)	90.2(2.8)
28	rimonabant	71.7(3.6)	72.3(1.8)	69.3(7.1)		91.7(1.6)		96.1(2.1)	99.9(3.7)
29	lidocaine	75.4(1.6)	74.7(2.9)	75.7(7.9)		80.0(1.4)		89.4(2.9)	95.5(2.4)
30	chlorpretadalafil	53.7(6.3)	72.9(5.2)	73.5(10)		69.5(7.5)		79.5(2.3)	89.8(5.7)
31	dimethylsildenafil	72.2(3.7)	54.6(2.6)	72.2(6.2)		79.8(5.2)		104.1(3.4)	97.5(2.1)
32	udenafil	70.7(3.6)	51.3(3.4)	70.6(8.5)		67.2(2.8)		85.7(1.8)	88.7(2.5)

总体而言,固体基质加标样品中32种化合物在3个加标浓度下的回收率略低于液体基质加标样品,可能是因为固体样品在50%甲醇水提取液中存在不溶物,产生的吸附作用造成提取不彻底。这就导致即使采用了基质匹配标准曲线进行定量,部分化合物回收率依然较低的情况。如硫代豪莫西地那非在固体基质中的回收率为23.4%~49.8%,低于液体基质的回收率64.9%~88.5%,但其在两种基质中的检出限均能达到0.02 mg/kg,即当供试品溶液质量浓度低至1 μg/L时,连续3针进样都能保证准确定性。另外药物非法添加的浓度均较高^[10,14⇓⇓⇓-18]^,综合考虑本方法的检出限和回收率能满足定性筛查的需要。

### 2.4 建立HRMS数据库及筛查方法

分别以50%甲醇水溶液稀释对照品溶液,得到2 mg/L或5 mg/L的32种化合物标准溶液,采用单针进样的方式分别在正负离子模式下进行Full MS/dd-MS^2^扫描,对比响应强度,选择合适的扫描模式、离子碎片精确质量数,在TraceFinder软件中输入至method development→compound database文件(.cdb)中。其中,高分辨一级质谱图用于化合物的筛查、鉴定和定量,二级质谱图用于化合物的确认。

调用上述.cdb文件,建立screening master method用于快速筛查定性,筛查条件的设置同1.4.3节。调用上述.cdb文件,建立quan master method用于阳性样品定量。关联较高浓度混合标准溶液的. raw文件(较高浓度以方便找到对应的化合物峰位置),确定阳性检出化合物的峰积分时间窗口、峰平滑点数,设置标准曲线类型为外标法,指定标准曲线点数和对应的浓度值,上述操作可批量进行。当目标峰、保留时间、碎片离子、同位素丰度比的筛查条件都通过时,TraceFinder软件的分析结果显示flag为绿灯,则可认为该化合物呈阳性。

本研究对TraceFinder软件筛查条件设置相关经验进行了总结,如下:保留时间是筛查分析中除了质量精度之外最重要的参数,其提供的是与质谱正交维度的信息,尤其对痕量物质的筛查至关重要。当自建数据库中的保留时间与样品采集方法相匹配时,建议将保留时间作为筛查条件。关于匹配模式的选择,选择identify时,意味着保留时间是化合物确证的必要条件,若此条件不满足要求,则不进行后续碎片离子和同位素丰度比的筛查;若选择confirm,意味着保留时间是化合物确证的一个可选条件,若此条件不满足要求,还是需要进行后续其他条件的匹配,比如碎片离子、同位素信息等。同位素信息在高分辨质谱中非常重要,是对一级精确质量数的有效补充,建议启用,但应考虑到目标物在复杂基质中含量很低时,同位素丰度比会更低,可能会影响同位素的匹配。另外在碎片离子的判定中,若在TraceFinder软件的筛查结果中未看到化合物碎片离子的判定结果,但在数据浏览器中可提取到碎片离子信息,则可将筛查条件中碎片离子的强度阈值进一步降低,查找相应的碎片离子信息。

### 2.5 实际样品检测

将Xcalibur软件采集的.raw原始数据文件导入至TraceFinder软件,调用screening master method进行快速定性筛查,同步采用外标标准曲线进行32种化合物的定量分析。

在48份样品中只有1份某公司生产的减肥茶检出大黄素成分,检出率2.08%,检出含量平均值为0.37 mg/kg。查配料表发现该样品含有决明子,而决明子富含大黄酚、大黄素、决明素^[19]^,加之检出浓度较低,可认为大黄素为内源性成分,并非非法添加。阳性样品中大黄素的提取离子色谱图、一级全扫描质谱图、二级质谱图及数据库对比图见图2、图3。大黄素的[M-H]^-^离子实测保留时间和理论保留时间偏差为0 s,一级质谱精确质量数实测值与理论值偏差为-1.08×10^-6^,两个二级碎片离子质量数偏差分别为5.11×10^-6^和0.9×10^-6^,上述结果均符合筛查方法中设置的控制条件,对应的筛查结果标记为绿灯,最终flag为绿灯,表明分析软件判定该样品中检出了大黄素。基于flag的标记情况,使用该HRMS数据库和筛查方法可进行快速筛查,严格的筛查条件降低了假阴性和假阳性的概率。

**图2:**
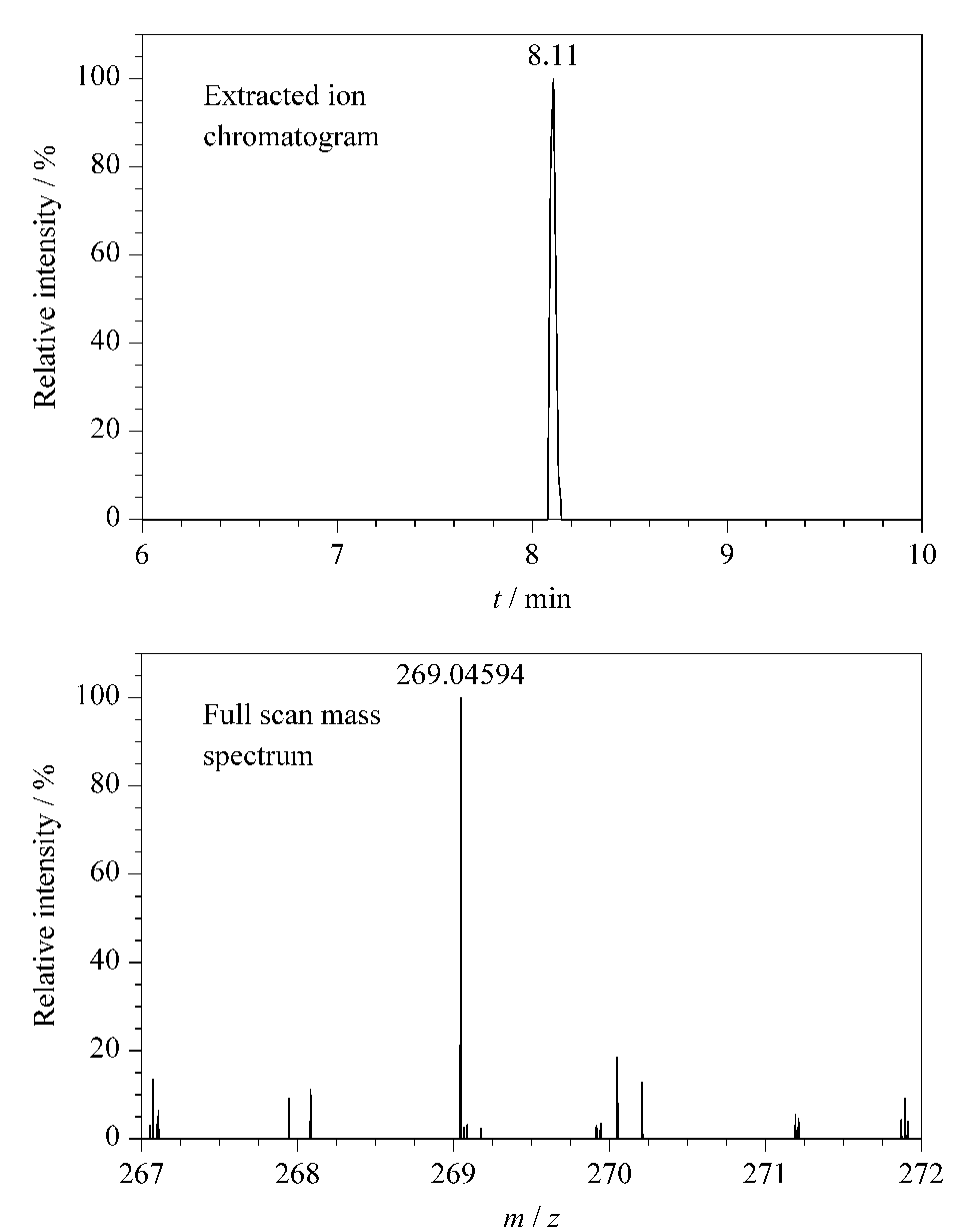
阳性样品中大黄素的提取离子色谱图和全扫描质谱图

**图3:**
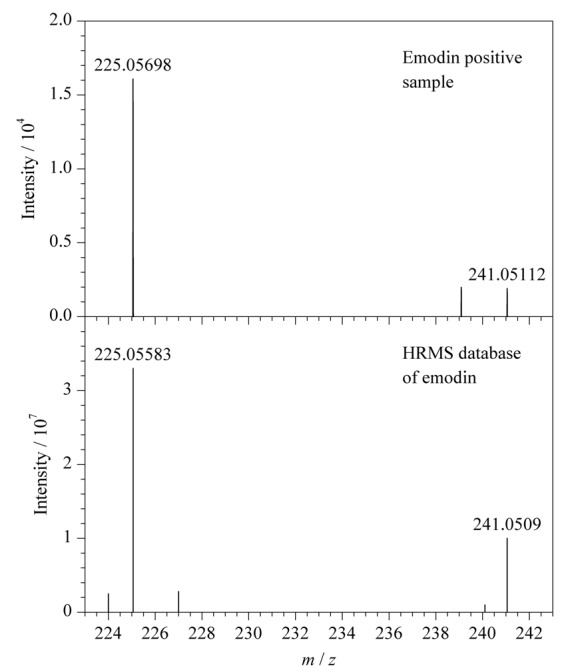
大黄素阳性样品的二级质谱图及其TraceFinder数据库谱图

## 3 结论

本研究基于实际检测工作需要建立了减肥和壮阳类保健食品中非法添加药物的快速筛查和确证方法,依靠TraceFinder软件建立的高分辨数据库,完成了快速定性筛查和定量分析,总结了数据库应用过程中的要点。本方法样品前处理简单,灵敏度高,筛查分析准确,自动化程度高,适用于同时筛查和测定减肥和壮阳类保健食品中的非法添加药物,具有很好的实际应用价值,已应用于日常检测工作。后续研究中在增加可筛查非法添加药物数量的同时应兼顾峰形和响应强度,同时继续深入研究基质效应对非法添加药物筛查的影响规律。
